# Challenges in interpreting leukocyte and nucleated red blood cell counts in neonatal hemolytic disease: A case report on hematology analyser performance

**DOI:** 10.5937/jomb0-60254

**Published:** 2026-01-28

**Authors:** Marina Jakšić, Lidija Banjac, Boban Banjac

**Affiliations:** 1 Department of Laboratory Diagnostics, Institute for Children's Diseases, and Centre for Science, Clinical Centre of Montenegro, Podgorica, Montenegro; 2 Faculty of Medicine, University of Montenegro, Podgorica, Montenegro; 3 Department of Neonatology, Institute for Children's Diseases, Clinical Centre of Montenegro, Podgorica, Montenegro; 4 Department of Diagnostic Imaging, Clinical Centre of Montenegro, Podgorica, Montenegro

**Keywords:** hemolytic disease of the newborn, nucleated red blood cells, white blood cell count, erythroblastosis, neonatal haematology, analyser performance, hemolitička bolest novorođenčeta, nukleusani eritrociti, ukupan broj leukocita, eritroblastoza, neonatalna hematologija, performanse analizatora

## Abstract

We present a case of hemolytic disease of the newborn (HDN) due to maternal alloimmunisation with anti-E and anti-c antibodies, resulting in severe anaemia, respiratory insufficiency, and hyperbilirubinemia in a term male neonate. Haematological evaluation using the automated analyser Sysmex XN-3100 (Sysmex Corporation, Kobe, Japan) yielded an erroneously elevated white blood cell (WBC) count of 1 6 3 x 1 0 / L , later manually corrected to 2 8 x 1 0 / L due to extreme nucleated red blood cell (NRBC) interference (&gt; 2,000 NRBCs per 100 WBCs). This case illustrates the analytical limitations of modern haematology analysers in neonates with pronounced erythroblastosis. It emphasises the essential role of manual peripheral blood smear review and interdisciplinary clinical-laboratory correlation in ensuring diagnostic accuracy.

## Abstract (sr)

Predstavljen je slučaj hemolitičke bolesti novorođenčeta (HBN) izazvane majčinom aloimunizacijom na anti-E i anti-c antitela, što je dovelo do teške anemije, respiratorne insuficijencije i hiperbilirubinemije kod terminskog muškog novorođenčeta. Hematološka analiza izvršena pomocu automatizovanog analizatora Sysmex XN -3100 (Sysmex Corporation, Kobe, Japan), pokazala je la no povišen broj leukocita u vrednosti od 1 6 3 x 1 0 /L, koji je kasnije manuelno korigovan na 2 8 x 1 0 /L, zbog ekstremne inter-feren CE nukleisanih eritrocita (više od 2.000 NRBC na 100 leukocita). Ovaj slučaj ilustruje analitička ograničenja savremenih hematoloških analizatora kod novorođenčadi sa izra-zenom eritroblastozom i naglašava ulogu pregleda razmaza periferne krvi i interdisciplinarne saradnje kliničara i laboratorije u obezbeđivanju dijagnostičke tačnosti.

## Introduction

Hemolytic disease of the newborn (HDN) is an immune-mediated condition in which maternal immunoglobulin G (IgG) antibodies target fetal red blood cell (RBC) antigens, crossing the placenta and leading to fetal hemolysis. Although Rh(D) incompatibility remains a classical aetiology, minor antigen incompatibilities - such as anti-E, anti-c, and anti-K - are increasingly recognised as significant contributors to perinatal morbidity and mortality [1] [2]. 

Erythroblastosis, marked by a pronounced elevation in nucleated red blood cells (NRBCs), is a haematological hallmark of HDN. In such cases, automated haematology analysers may be prone to misclassification errors, particularly when the NRBC burden exceeds standard flagging thresholds. This may lead to pseudoleukocytosis, complicating differential diagnoses such as neonatal sepsis [3]. The case presented herein highlights these limitations and the diagnostic implications of extreme NRBCemia on automated haematology platforms.

## Results

A term male neonate was admitted to the neonatal intensive care unit in the fourth hour of life due to respiratory distress and an oxygen saturation of 68%. Clinical examination revealed pallor, jaundice, tachypnea, tachycardia, and hepatosplenomegaly (2.5 cm below the costal margin). The umbilical cord was thickened and discoloured yellow-green, suggestive of intrauterine stress. The mother's obstetric history included a prior pregnancy complicated by fetal haemolytic anaemia. An exchange transfusion was performed in response to severe anaemia. Initial laboratory findings showed severe anaemia, indirect hyperbilirubinemia and leukocytosis (lymphocytosis) (Table 1).

**Table 1:** Comparison of general data [x̄±s, n(%)].

Parameter	Result
Hemoglobin	70 g/L
WBC (analyser)	163x10^9^/L
Neutrophils	14.2x10^9^/L
Eosinophils	1.5x10^9^/L
Basophils	1.5x10^9^/L
Lymphocytes	120x10^9^/L
Monocytes	25x109/L
Indirect bilirubin	237 μmol/L
Direct antiglobulin test (DAT)	Positive (3+)

Blood typing was performed with the following results: neonate: A Rh(D)+, C+, c+, E + , e+, K-; mother: A Rh(D)+, CC, D-, ee, K-; maternal alloantibodies: anti-E and anti-c (IAT positive). A whole blood sample was collected in an EDTA tube, and a complete blood count was performed using a Sysmex XN-3100 analyser (Sysmex Corporation, Kobe, Japan), employing impedance and fluorescence-based flow cytometry. The analyser reported an erroneously high white blood cell (WBC) count of 163x0^9^/L, without generating any suspect flag. A peripheral blood smear was conducted following the suspicion. Highly skilled laboratory personnel count 100 nucleated cells on a Wright-stained peripheral blood smear to get the total WBC count. Manual review revealed a corrected WBC count of approximately 28x10/L and an NRBC count exceeding 2,000 per 100 WBCs on peripheral blood smear.

## Discussion

Modern haematology analysers such as the Sysmex XN-3100 typically flag and enumerate NRBCs using advanced scatter and fluorescence parameters. However, in cases of extreme erythroblastosis, NRBCs may be misclassified due to overlap in cellular size and fluorescence characteristics, leading to pseudoleukocytosis [4] [5]. Due to the automated method's absence of NRBC enumeration and consequent analytical error, the analyser in this instance greatly overstated the WBC count. Therefore, if not carefully examined, this serious interference might have led to needless antibiotic treatment and additional testing. Elevated NRBCs in neonates can result from hemolysis, chronic intrauterine hypoxia, growth restriction, or maternal conditions such as hypertension and diabetes [6] [7]. While mild NRBCemia is common, extreme values exceeding 2,000 per 100 WBCs strongly suggest a pathological process and warrant thorough review and clinical correlation [3].

This instance illustrates how automated haematology analysers' diagnostic flaws may impact neonatal patients with haemolytic disease. Significant NRBC interference can result in inaccurate automated WBC counts, leading to misdiagnosis and incorrect treatment decisions. Manual peripheral blood smear review and close interdisciplinary communication are still crucial in cases when suspicions are raised to ensure patient safety and diagnostic accuracy.

## Dodatak

### Funding

No specific funding was received for this study.

### Ethics approval and consent to participate

The data used in this report were collected as part of routine clinical care.

### Author contributions

Marina Jaksic conceptualised, collected, and analysed data and wrote the manuscript. Lidija Banjac & Boban Banjac prepared a literature review and helped in writing the manuscript.

### Conflict of interest statement

All the authors declare that they have no conflict of interest in this work.
